# Effects and Safety of an Oral Adsorbent on Chronic Kidney Disease Progression: A Systematic Review and Meta-Analysis

**DOI:** 10.3390/jcm8101718

**Published:** 2019-10-17

**Authors:** Ying-Chun Chen, Mei-Yi Wu, Ping-Jen Hu, Tzu-Ting Chen, Wan-Chen Shen, Wei-Chiao Chang, Mai-Szu Wu

**Affiliations:** 1 Department of Pharmacy, Shuang Ho Hospital, Taipei Medical University, New Taipei City 23561, Taiwan; 12556@s.tmu.edu.tw (Y.-C.C.); 16323@s.tmu.edu.tw (W.-C.S.); 2 Division of Nephrology, Department of Internal Medicine, School of Medicine, College of Medicine, Taipei Medical University, Taipei 11031, Taiwan; e220121@gmail.com; 3 Division of Nephrology, Department of Internal Medicine, Shuang Ho Hospital, Taipei Medical University, New Taipei City 23561, Taiwan; 4 Institute of Epidemiology and Preventive Medicine, College of Public Health, National Taiwan University, Taipei 10617, Taiwan; uniquepapa@gmail.com; 5 Department of Primary Care Medicine, Shuang Ho Hospital, Taipei Medical University, New Taipei City 23561, Taiwan; 6 Division of Gastroenterology and Hepatology, Department of Internal Medicine, Taitung MacKay Memorial Hospital, Taitung 95054, Taiwan; a801891@hotmail.com; 7 Division of Gastroenterology, Department of Internal Medicine, Shuang Ho Hospital, Taipei Medical University, New Taipei City 23561, Taiwan; 8 Master Program in Clinical Pharmacogenomics and Pharmacoproteomics, School of Pharmacy, Taipei Medical University, Taipei 11031, Taiwan; 9 Department of Clinical Pharmacy, School of Pharmacy, Taipei Medical University, Taipei 11031, Taiwan; 10 Integrative Research Center for Critical Care, Wan Fang Hospital, Taipei Medical University, Taipei 11696, Taiwan; 11 Department of Medical Research, Shuang Ho Hospital, Taipei Medical University, New Taipei City 23561, Taiwan

**Keywords:** AST-120, Kremezin, chronic kidney disease, systematic review, meta-analysis

## Abstract

Background: AST-120 (Kremezin), which is an oral spherical carbon adsorbent, has been reported to have the potential for retarding disease progression in patients with chronic kidney disease. We aimed to evaluate its efficacy and safety in this study. Methods: We systematically searched for randomized controlled trials published in PubMed, Embase, and Cochrane databases. The primary outcomes were the renal outcome and all-cause mortality, and the change in serum indoxyl sulfate (IS) levels. The safety outcome was also evaluated in terms of reported major adverse events. A random-effects model was used when heterogeneity was expected. Results: Eight studies providing data for 3349 patients were included in the meta-analysis. The risk ratio of renal outcome and all-cause mortality were 0.97 (95% CI: 0.88–1.07; 6 trials) and 0.94 (0.73–1.20; 5 trials), respectively. Furthermore, the weighted mean change in IS levels from baseline to the end of the study was −0.28 mg/dL (95% CI: −0.46 to −0.11; 4 trials). Conclusions: This study provides evidence that AST-120 can effectively lower IS levels but still controversial in terms of slowing disease progression and all-cause mortality. Except for dermatological events, the incidence of adverse events did not differ significantly between the AST-120 and placebo groups.

## 1. Introduction

Chronic kidney disease (CKD) is a global health concern [1] where many patients with the disease advance to end-stage renal disease (ESRD), which requires dialysis or kidney transplantation, increases mortality, and reduces quality of life [1,2]. According to one estimate, by 2030, patients with ESRD will account for 5.4 million people on dialysis [3], making early management of CKD and its risk factors a crucial clinical concern. Current guidelines for the management of CKD focus on controlling factors that can accelerate CKD progression, such as hypertension and diabetes [4,5].

AST-120 is an orally administered adsorbent, which consists of spherical particles 0.2–0.4 mm in diameter. It is predominantly composed of carbon [6] and can adsorb uremic toxins and precursors, such as indoxyl sulfate (IS), causing their subsequent excretion in feces [6,7]. AST-120 was shown to suppress oxidative stress in uremic rats [8] and has been reported to effectively slow the progression of glomerular sclerosis, tubulointerstitial fibrosis, and proximal tubular hypertrophy [9,10]. As a pharmaceutical agent, AST-120 was first approved in Japan in 1991 to alleviate uremic symptoms and delay the need for dialysis in patients with CKD [6]. It was later approved in Korea, Taiwan, and the Philippines for treating CKD patients.

Over the past two decades, the effects of AST-120 have been evaluated in patients with CKD in many prospective clinical trials and retrospective studies. However, the renoprotective effects of AST-120 are still relatively unknown. Furthermore, it is also unclear whether current evidence supports the recommended use of AST-120 in CKD patients. In the present study, we evaluated AST-120’s safety and efficacy in retarding CKD progression. To do so, we conducted a systematic review and meta-analysis of published literature with a preplanned study protocol, well-designed search strategy, and a critical assessment of comprehensive studies.

## 2. Materials and Methods

### 2.1. Search Strategy and Selection Criteria

We searched the Pubmed, Embase, and Cochrane CENTRAL databases from their inception dates up to 31 July 2018 using the free-text key words, MeSH terms, or Emtree terms: “AST-120” or “Kremezin” and “chronic kidney disease.” Unpublished studies were searched on ClinicalTrials.gov, and the references cited in the selected studies were reviewed to obtain additional relevant literature. The investigators reviewed the titles and abstracts of the retrieved studies to exclude duplicate studies, irrelevant studies, and studies that did not meet the eligibility criteria. The full text of potentially relevant studies was carefully reviewed, and the studies were included if: (1) patients with stages 3–5 CKD were enrolled, (2) the effects of AST-120 were evaluated, (3) patients were randomized into a group receiving AST-120 and another receiving a placebo or conventional treatment in the trial, and (4) one or more primary or secondary outcomes were reported. A study was excluded if it: (1) did not report outcomes of interest clearly, (2) did not provide sufficient information to extract or calculate treatment effects, (3) was not a randomized controlled trial (RCT), or (4) evaluated the same or overlapping patient cohorts as one or more other studies. Our systematic review has been accepted by PROSPERO, an online international prospective register of systematic reviews, which is funded by the National Institute for Health Research (CRD42019136927).

### 2.2. Primary and Secondary Outcomes

The primary outcomes were a composite of renal outcomes, all-cause mortality, and the change in serum IS levels. The composite of renal outcomes comprised the doubling of serum creatinine levels, increase in serum creatinine levels to ≥6.0 mg/dL, 50% reduction in estimated glomerular filtration rate (eGFR), initiation of dialysis, and initiation of kidney transplantation. Safety of AST-120 was evaluated using the risk of constipation, diarrhea, gastrointestinal disorders, and dermatological events. 

### 2.3. Data Extraction and Risk of Bias Assessment

Two investigators (Mei-Yi Wu and Ying-Chun Chen) independently reviewed the included trials and extracted relevant data. After eliminating duplicate records, the two investigators independently examined the titles and abstracts to exclude clearly irrelevant studies. Next, the investigators independently retrieved and examined full texts of potentially relevant studies to determine whether the studies met the inclusion criteria. The investigators made a final decision regarding the inclusion of eligible studies and extracted information, including the name of the first author, publication year, country, study design, number of total patients and number in each arm, experimental drugs administered, population, age, duration of follow-up, baseline serum creatinine levels, composite of renal outcomes, all-cause mortality, change in serum IS levels, constipation, diarrhea, gastrointestinal disorders, and dermatological events. Because the mean change in serum IS levels from baseline was unavailable for each patient, the mean change in the IS levels in each treatment was calculated by subtracting the final mean from the baseline mean. When the standard deviation of the change from baseline in each treatment was not available, we estimated the missing standard deviation by using an imputed value for measures with a correlation coefficient of 0.5. Moreover, two reviewers independently assessed the risk of bias, including the selection bias, performance bias, detection bias, attrition bias, reporting bias, and other bias, by using the Cochrane risk of bias tool for randomized trials [11]. 

### 2.4. Data Synthesis and Analysis

The weighted mean difference for the change in serum IS levels and risk ratios (RRs) for the composite of renal outcomes, all-cause mortality, and adverse effects among different treatments were calculated. A fixed-effect model with a common treatment effect and a random-effects model with a heterogeneous treatment effect across studies were used. Pooled estimates of the weighted mean difference were computed using the Mantel–Haenszel test and estimates of RRs were made with the inverse variance method. The precision of effect sizes was estimated using 95% confidence intervals (CIs). Statistical heterogeneity was assessed using the I^2^ statistic, with I^2^ values of >50% indicating substantial heterogeneity [12]. Funnel plots were used to determine publication bias regarding the end points. In this study, zero correlation was assumed between the change-from-baseline outcome measures when paired analyses were not available [13]. Moreover, sensitivity analysis was conducted to resolve the issue of synthesizing data from crossover trials into meta-analyses. All statistical analyses were performed using the statistical package, Review Manager, version 5.3 (Cochrane Collaboration, Oxford, England).

## 3. Results

### 3.1. Subsection

#### 3.1.1. Study Characteristics

**Figure 1:**
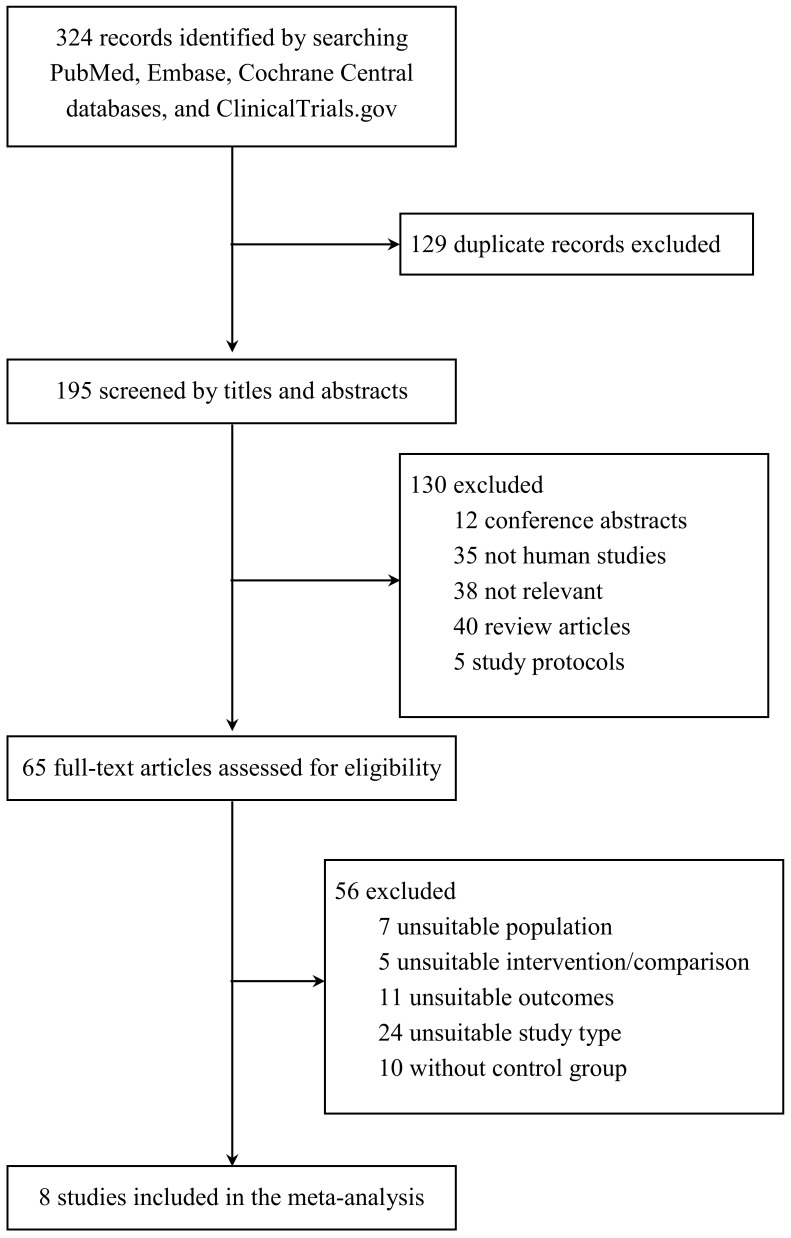
Flowchart of the literature search and trial selection.

In our analysis, we included eight studies that included a total of nine RCTs [14,15,16,17,18,19,20,21]. A flowchart summarizing the screening and selection process for the studies is shown in Figure 1. Our initial search strategy yielded 324 citations, but 129 were excluded as duplicate records. One hundred and thirty citations were excluded after screening the titles and abstracts. We then retrieved the full texts of 65 studies. Of these 65, we excluded studies that were not RCTs, studies that did not have a control group, and those in which the population, intervention, and outcomes did not meet the requirements of our meta-analysis. Finally, we included eight studies with nine RCTs in our analysis. The nine RCTs were published between 1997 and 2016 and had sample sizes ranging from 26 to 1007; the total number of participants across the studies was 3349 (Table 1). All RCTs recruited patients with stage 3–5 CKD, and the mean patient ages ranged from 54.4 to 69.3 years. The AST-120 dosages, ranging from 2.7 to 9 g/day, were adjusted according to various protocols. The follow-up lengths in the included trials ranged from 7 days to 3 years. Serum creatinine levels at baseline varied widely across studies, ranging from 2.36 to 5.75 mg/dL.

#### 3.1.2. Study Quality

**Figure 2:**
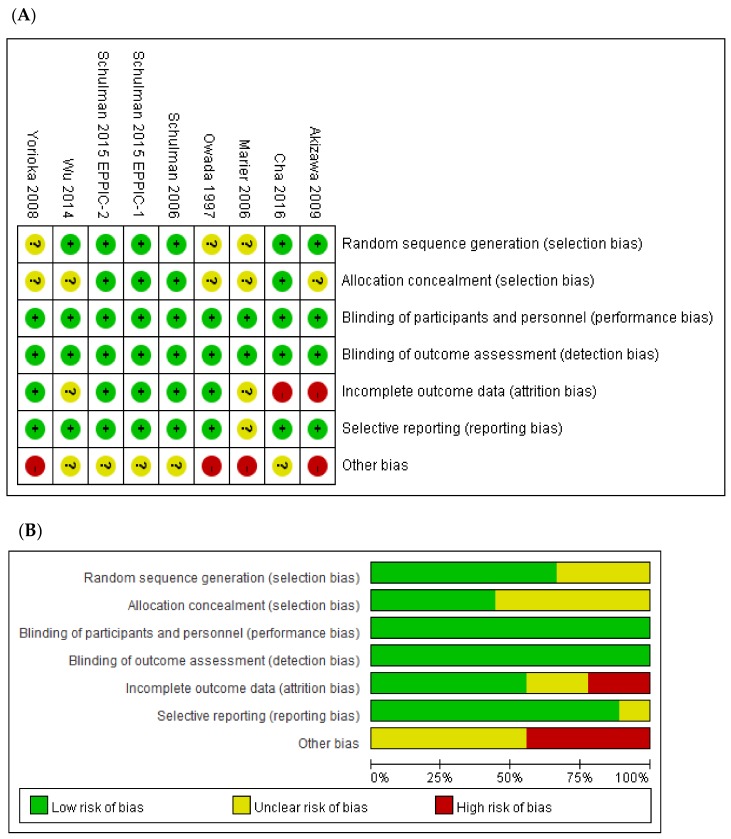
Risk of bias. (**A**) Risk of bias summary; (**B**) Risk of bias graph.

The risk of bias assessment is shown in Figure 2. Six RCTs clearly documented the randomization process [14,15,18,19,20], and four RCTs provided detailed descriptions of allocation concealment [15,18,19]. An open-label design was used in 44.4% of the included studies [14,15,20,21], which could have introduced investigator or subject bias in the reporting of adverse effects. However, the primary outcomes were all laboratory values and were therefore unlikely to be affected by a lack of blinding. Two RCTs were considered to be at a high risk of attrition bias due to per-protocol analyses or differences in the proportion of incomplete outcome data across groups [14,15]. An RCT was considered to be at high risk of other bias if the sample size calculation differed from the real data [14]. Additionally, RCTs with small sample sizes were considered to have a high risk of other bias [16,17,21].

#### 3.1.3. Publication Bias

We did not assess the publication bias of the nine studies using the funnel plot asymmetry test because the test is unable distinguish chance from actual asymmetry when fewer than 10 studies are tested.

#### 3.1.4. Primary Outcomes

##### Composite of Renal Outcomes

**Figure 3:**
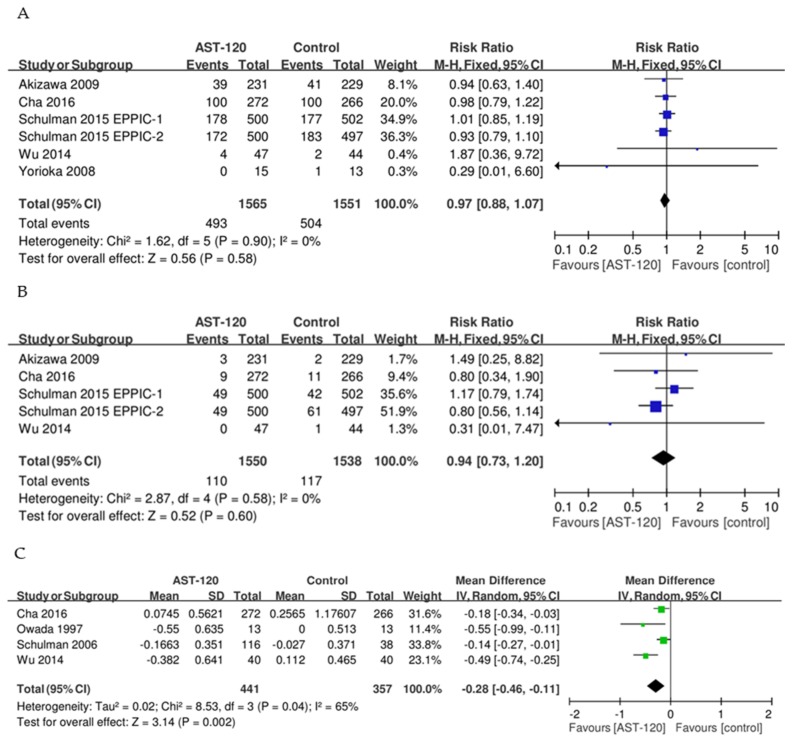
Result of primary outcomes for comparison between AST-120 and control groups. (**A**) Composite of renal outcome; (**B**) All-cause mortality; (**C**) Change in serum indoxyl sulfate levels.

The meta-analysis for the composite of renal outcomes included six trials [14,15,19,20,21] with a total of 3116 participants (Figure 3A). Using a fixed-effect model, we found that the RR of the composite of renal outcomes was 0.97 (95% CI: 0.88–1.07; heterogeneity I^2^: 0%, *p* = 0.9). Thus, our meta-analysis revealed that the AST-120 groups did not significantly differ from placebo and conventional treatment groups. We also determined that the RR of the composite of renal outcomes computed in the sensitivity analysis (after excluding crossover trials) was the same as our main result.

##### All-Cause Mortality

Five studies [14,15,19,20] with a total of 3088 participants provided data on all-cause mortality (Figure 3B). We used a fixed-effect model to estimate the RR for this outcome. The RR of all-cause mortality was 0.94 (95% CI: 0.73–1.20) with low heterogeneity (I^2^: 0%, *p* = 0.58). Thus, our meta-analysis also showed no significant difference in all-cause mortality between the AST-120 groups and placebo or conventional treatment groups. The RR of all-cause mortality computed in the sensitivity analysis (after excluding the crossover trials) was the same as the main result.

##### Change in Serum IS Levels

In a meta-analysis of four studies, which included a total of 798 participants, the weighted mean change in serum IS levels from baseline to the end of the study was −0.28 (95% CI: −0.46 to −0.11) in patients treated with AST-120 compared with those receiving placebo or conventional treatment (Figure 3C). We assumed that the treatment effect was heterogeneous across studies, so we used a random-effects model to estimate the weighted mean change in serum IS levels (heterogeneity I^2^: 65%, *p* = 0.04). In the sensitivity analysis, after the exclusion of crossover trials, the weighted mean change in serum IS from baseline to the end of the study was −0.19 (95% CI: −0.33 to −0.06) in patients treated with AST-120 compared with those who received placebo or conventional treatment.

#### 3.1.5. Secondary Outcomes

**Figure 4:**
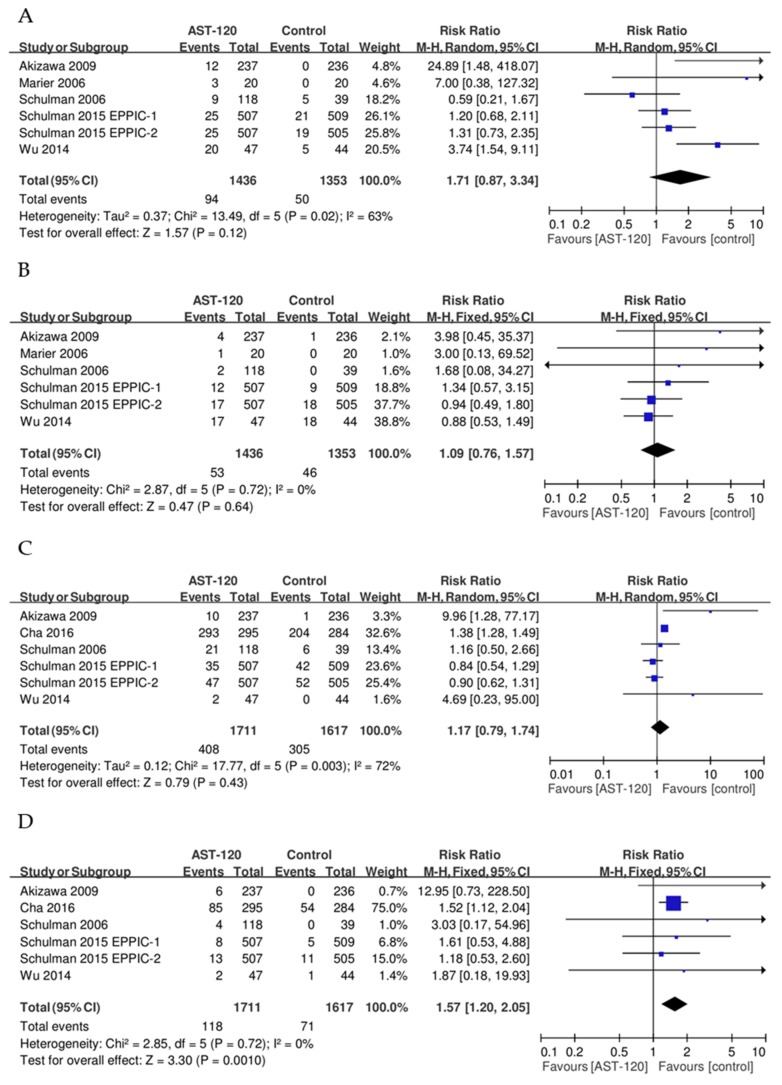
Result of secondary outcomes for comparison between AST-120 and control groups. (**A**) Constipation; (**B**) Diarrhea; (**C**) Gastrointestinal disorders; (**D**) Dermatological events.

In this meta-analysis, the risks of constipation, diarrhea, and gastrointestinal disorders did not differ significantly between patients treated with AST-120 and those treated with placebo or conventional drugs. Compared with individuals treated with placebo or conventional drugs, the RR of dermatological events among patients treated with AST-120 was 1.57 (95% CI: 1.20–2.05). Details of the comparisons between AST-120 and the placebo or conventional groups are provided in Figure 4. The results of our sensitivity analysis, after the exclusion of crossover trials, were identical to the main result.

## 4. Discussion

The present meta-analysis included eight studies with nine RCTs, representing a total of 3349 patients with stage 3–5 CKD. The analyzed studies collectively showed that patients with CKD who received AST-120 did not register a considerable improvement in renal outcome and all-cause mortality, but AST-120 did reduce the level of serum IS. We also found that the risks of constipation, diarrhea, and gastrointestinal disorders did not differ significantly between patients treated with AST-120 and control groups; however, dermatological events were more common in patients treated with AST-120 than in those treated with placebo or conventional drugs.

CKD results in the accumulation of metabolic waste products that are normally cleared by the kidneys. Studies have demonstrated that the accumulation of uremic toxins, such as IS and p-cresyl sulfate, induces free radical production in renal tubular cells and glomerular mesangial cells [22,23], which then accelerates CKD progression [24]. Among uremic toxins, IS is the most well studied. It is formed from indole, a tryptophan metabolite, which is synthesized by intestinal bacteria and absorbed from the intestine into the blood. Once in circulation, indole may be sequentially converted to indoxyl and IS in the liver [25]. One possible mechanism that may allow AST-120 to reduce IS is that it adsorbs indole in the intestine and causes the indole to be excreted in the feces, thereby limiting the bioavailability of the IS precursor [7,26].

In contrast to the findings from our meta-analysis, AST-120 has been shown to delay CKD progression in some studies, most of which have been conducted in Japan [27,28,29]. In a Japanese phase III trial [29], patients who received AST-120 treatment for 24 weeks were significantly more likely to show improvements in the change of reciprocal of the creatinine level compared to the placebo group (43% and 24% in the AST and placebo groups, respectively, *p* < 0.01). In addition, the time to achieve the primary endpoints of the doubling of serum creatinine levels or the initiation of dialysis differed significantly between the two groups [29].

The difference between our meta-analysis results and the Japanese phase III trial may have several possible explanations. First, the discrepancy may be attributable to differences between actual and estimated event curves. For example, AST-120 was not efficacious in delaying CKD progression in two randomized double-blind phase III trials (EPPIC-1 and EPPIC-2) [19]; however, in the placebo groups of these studies, the estimated median time to the primary endpoint of CKD progression was 124 weeks, whereas the actual mean times were 189.0 and 170.3 weeks for the EPPIC-1 and EPPIC-2 trials, respectively. The delays suggest that the controls in these studies progressed more slowly than expected. Nevertheless, post-hoc studies demonstrated that AST-120 patients with progressive CKD [30] and higher compliance [31] potentially delay the time to the primary end point. Moreover, the eGFR level was more stable in the AST-120 group, especially in patients with diabetic nephropathy [32].

Second, regional differences in the decision to initiate dialysis could have contributed to the differences in renal outcomes. Patients in Western countries typically had higher mean eGFR values at the initiation of dialysis than did patients in Asian countries [33]. This difference may reflect differing evaluations of disease progression between Western and Asian practitioners. Third, variations in diet and nutritional status between countries may have affected the results. Despite the unconfirmed role of nutrition in CKD, some reviews have suggested that unrestricted protein intake may increase the risk of disease progression [34]. Thus, our meta-analysis may have failed to identify the clinical benefits of AST-120 based on technical or regional issues that influenced the results of phase III trials.

According to previous publications, we expected that AST-120 should significantly reduce serum IS levels [18]. In a multicenter RCT on patients with moderate to severe CKD, 12 weeks after receiving AST-120 treatment, patients treated with 9.0 g and 2.7 g of AST-120 showed 39.3% and 2.6% reductions, respectively, in serum IS levels [18]. The theoretical maximal amount of creatinine that can be absorbed in the gastrointestinal tract after the administration of 9.0 g/day of AST-120 is approximately 10 mg/g [34,35]. In our meta-analysis, the daily AST-120 dosage in the included studies ranged from 2.7 to 9.0 g, and we saw reduced serum IS levels, which is consistent with the findings of previous studies [35]. 

Decreasing serum IS levels may be beneficial for cardiovascular outcomes [36,37,38]. Barreto et al. demonstrated that serum IS levels were positively and significantly associated with aortic calcification and pulse wave velocity [36]. Sato et al also reported that in patients with a higher IS level, there was a higher proportion of left ventricular dysfunction [39]. In previous studies, IS has been implicated in the pathogenesis of at least six phenotypes of cardiovascular disease [40], including atherosclerosis [37], arteriosclerosis [41], congestive heart failure [38], arrhythmia [42,43], peripheral arterial disease [44], and vascular thrombosis [45]. Several studies revealed that high serum IS levels independently predicted overall mortality [36,46]. However, in our study, all-cause mortality was not correlated with serum IS level.

Because AST-120 is not absorbed into the bloodstream or tissue, it is not expected to exert major systemic effects [6,18]. The most commonly reported treatment-related adverse events for AST-120 include mild to moderate constipation, diarrhea, and flatulence, which are all effects on the gastrointestinal system [16,18]. Several studies have also investigated relatively rare adverse events, such as pruritus, poor appetite, and nutritional problems; in most previous studies and reviews, the incidence of non-gastrointestinal adverse events did not differ significantly between the AST-120 and placebo groups [15,18,19].

Compared with previous meta-analyses, our study has several strengths. First, all included RCTs were critically assessed, and the overall risk of bias was lower than that reported in previous meta-analyses. Second, we considered all relevant outcomes (namely all-cause mortality, change in serum IS levels, and incidence of adverse gastrointestinal and dermatological effects). Third, we comprehensively searched multiple databases in all languages; therefore, we are unlikely to have missed a substantial number of relevant studies. 

Our study also has several limitations. First, the progression rate for CKD is usually estimated from the slope of a plot with the reciprocal of creatinine versus time. However, the actual curves may differ considerably from estimated curves. Second, differences in population characteristics, region, diet and nutrition status, AST-120 dosage, renal function status, and concurrent medications in the included studies could have contributed to the observed heterogeneity in results. Third, the length of follow-up varied across studies, resulting in potentially significant variations in the incidence of adverse effects.

## 5. Conclusions

Although AST-120-treated patients had reduced serum IS levels, they did not exhibit considerable improvements in renal outcome and all-cause mortality. Except for dermatological events, the incidences of adverse events in patients treated with AST-120 were similar to those in placebo groups. Because of limitations and bias in previous studies, possible clinical benefits, such as the alleviation of uremic symptoms, were not assessed in our study. Additional large-scale RCTs with longer follow-up durations and standardized outcomes are still necessary to clarify the clinical efficacy of AST-120.

## Figures and Tables

**Table 1:** Characteristics of included studies.

Study	Country/Region	Population	Intervention/Comparison	Duration	No. of Patients (% of male)	Age	Baseline sCr
Akizawa 2009 [14]	Japan	CKD stages 3–5	I: AST-120 6 g/day	56 weeks	I: 231 (34.6)	I: 62.9 ± 13	I: 2.66 ± 1.03
			C: LPD and RASi		C: 229 (31.9)	C: 63.3 ± 11	C: 2.65 ± 1.05
Cha 2016 [15]	Korea	CKD stages 3 or 4	I: AST-120 6 g/day	36 months	I: 272 (66.9)	I: 56.7 ± 13.3	I: 2.82 ± 0.66
			C: Placebo + standard care		C: 266 (68.0)	C: 56.8 ± 13.2	C: 2.84 ± 0.70
Marier 2006 [16]	USA	1.5 ≤ sCr ≤ 6.0 mg/dL	I: AST-120 9 g/day	7 days	I: 20	58	I: 2.36 ± 1.07
			C: Placebo	(Washout: 9 days)	C: 20		C: NA
Owada 1997 [17]	Japan	CKD (sCr 3–8.6 mg/dL)	I: AST-120 6 g/day + LPD (0.6 g/kg)	12–24 months	I: 13	NA	I: 5.75 ± 0.99
			C: LPD (0.6 g/kg)		C: 13		C: 4.77 ± 1.50
Schulman 2006 [18]	USA	sCr 3–6 mg/dL	I_1_: AST-120 9 g/day	12 weeks	I_1_: 39 (76.9)	I_1_: 69.3 ± 13.93	I_1_: 4.33 ± 0.87
			I_2_: AST-120 6.3 g/day		I_2_: 40 (55.0)	I_2_: 66.3 ± 10.24	I_2_: 4.47 ± 0.89
			I_3_: AST-120 2.7 g/day		I_3_: 39 (76.9)	I_3_: 59.6±13.83	I_3_: 4.35 ± 0.97
			C: Placebo		C: 39 (64.1)	C: 63.1 ± 12.94	C: 4.58 ± 1.04
Schulman 2015 [19]	USA, Latin America, Europe	CKD stages 3–5	I: AST-120 9 g/day	I: 91.0 ± 50.3 weeks	I: 500 (61.8)	I: 56.3 ± 14.9	I: 3.09 ± 0.88
EPPIC-1			C: Placebo	C: 92.6 ± 52.6 weeks	C: 502 (79.9)	C: 55.6 ± 14.9	C: 3.10 ± 0.84
Schulman 2015 [19]	USA, Latin America, Europe	CKD stages 3–5	I: AST-120 9 g/day	I: 94.1 ± 49.9 weeks	I: 500 (54.6)	I: 54.4 ± 15.5	I: 3.06 ± 0.87
EPPIC-2			C: Placebo	C: 87.8 ± 50.6 weeks	C: 507 (55.5)	C: 55.5 ± 14.6	C: 3.18 ± 0.90
Wu 2014 [20]	Taiwan	CKD stage 5	I: AST-120 6 g/day + Mircera 1.2 mcg/kg Q4W	12 weeks (Washout: 4 weeks)	51 (31.4) ITT	61.26 ± 11.49	I: 5.48 ± 2.31
		Hb < 10 g/dL	C: Mircera* 1.2 mcg/kg Q4W		40 (35) PP		C: 5.14 ± 2.64
Yorioka 2008 [21]	Japan	sCr 1.5–5.0	I: AST-120 6 g/day + conventional	12 months	I:15 (73.3)	I: 61.7 ± 12.6	I: 2.4 ± 0.8
		eGFR 15–60	C: Conventional (LPD 0.8 g/kg and RASi)		C:13 (58.5)	C: 59.7 ± 8.9	C: 2.7 ± 0.8

Abbreviations: C—Comparison; CKD—Chronic kidney disease; I—Intervention; ITT—Intention to treat; LPD—Low-protein diet; PP—Per protocol; RASi—Renin-angiotensin system inhibitor; sCr—serum creatinine. * Methoxy polyethylene glycol-epoetin beta (MIRCERA, Roche).
